# Functional and Brain Activation Changes Following Specialized Upper-Limb Exercise in Parkinson’s Disease

**DOI:** 10.3389/fnhum.2019.00350

**Published:** 2019-10-15

**Authors:** Luca Valerio Messa, Federica Ginanneschi, Davide Momi, Lucia Monti, Carla Battisti, David Cioncoloni, Barbara Pucci, Emiliano Santarnecchi, Alessandro Rossi

**Affiliations:** ^1^Department of Medical, Surgical and Neurological Sciences, University of Siena, Siena, Italy; ^2^Siena Brain Investigation and Neuromodulation Lab, Department of Medicine, Surgery and Neurological Sciences, University of Siena, Siena, Italy; ^3^Department of Neuroscience, Imaging and Clinical Sciences, University of Chieti-Pescara, Chieti, Italy; ^4^Unit of Neuroimaging and Neurointervention, Department of Neurological and Neurosensorial Sciences, Azienda Ospedaliera Universitaria Senese, Siena, Italy; ^5^U.O.P. Professioni della Riabilitazione, Azienda Ospedaliera Universitaria Senese, Siena, Italy; ^6^Berenson-Allen Center for Non-Invasive Brain Stimulation, Beth Israel Deaconess Medical Center, Harvard Medical School, Boston, MA, United States; ^7^The Center for Complex Network Research, Department of Physics, Northeastern University, Boston, MA, United States

**Keywords:** ASL, EquiTest, forced exercise, fMRI, resting-state fMRI, Parkinson’s disease

## Abstract

For the management of Parkinson’s disease (PD), the concept of forced exercise (FE) has drawn interest. In PD subjects, the FE executed with lower limbs has been shown to lessen symptoms and to promote brain adaptive changes. Our study is aimed to investigate the effect of an upper-limb exercise, conceptually comparable with the FE, in PD. Upper-limb exercise was achieved in a sitting position by using a specially designed device (Angel’s Wings^®^). Clinical data, computerized dynamic posturography, magnetic resonance imaging (MRI) (resting-state MRI and arterial spin labeling), and neuropsychological tests were used before and after 2 months’ exercise training. We found a significant long-lasting improvement in Unified Parkinson Disease Rating Scale (UPDRS)-III and cognitive scales, along with improvement in balance and postural control (better alignment of the gravity center and improvement in weight symmetry and in anticipatory motor strategies). Computerized dynamic posturography pointed out an enhanced central ability to integrate the vestibular signals with afferents from other sensory systems. Neuroimaging analyses after 2 months’ exercise training showed, with respect to pretraining condition, many changes. An increase of the cerebral blood flow was evident in the left primary motor cortex (M1), left supplementary motor cortical area, and left cerebellar cortex. The bilateral globus pallidus showed an increased functional connectivity to the right central operculum, right posterior cingulate gyrus, and left sensorimotor cortex. Seed-to-voxel analysis demonstrated a functional connectivity between M1 and the left superior frontal gyrus. Left crus II showed strengthened connections with the left pre-rolandic area, left post-rolandic area, and left supramarginal area. These findings likely reflect compensatory mechanisms to the neuropathological hallmark of PD. Overall, our results show that this upper-limb exercise model, conceptually comparable with the FE already tested in the lower limbs, leads to a global improvement (involving non-exercised limbs) likely consistent with the functional changes observed in the central nervous system.

## Introduction

Parkinson’s disease (PD) is characterized by motor and non-motor symptoms. Among motor symptoms, rest tremor, rigidity, and bradykinesia are mainly dependent on dopaminergic nigrostriatal denervation, whereas posture, balance, and gait dysfunctions are ascribed to degeneration of non-dopaminergic pathways (David et al., 2012). Balance control is critical for moving in safety and adapting to the environment, and balance disorders significantly contribute to impairment and disability in these patients (David et al., 2012). Balance control depends on an accurate regulation of tonic and phasic muscular activities that are made mechanically, without conscious awareness. The sensorimotor control of posture involves a complex integration of multisensory inputs that result in a final motor adjustment process. In PD subjects, the components of this system may be malfunctional, rendering postural instability one of the most disabling symptoms of PD. In addition, the characteristic forward position of the head altering spinal alignment is *per se* a biomechanical problem for balance control. Indeed, because the necessary condition for equilibrium is that all the linear and rotatory forces acting on the body must be balanced (i.e., theoretically, the sum of all the forces acting on the body must equal zero), the abovementioned static postural abnormalities generate antero-inferior forces that should be actively counterbalanced. In fact, there is evidence of loss of the ability to extend the trunk and head early in PD owing to extensor muscle weakness (Grabli et al., 2012).

Several rehabilitative approaches have been proposed to ameliorate posture and balance in PD. Although evidence exists of short-term, significant benefit for balance and clinician-rated disability in PD, it is not enough to sustain or refute the superiority of an approach over another. Nevertheless, physical exercise is considered as the basic element of all the neuro-rehabilitative treatment in PD patients (Abbruzzese et al., 2016). It has been suggested that contradictory results in rehabilitative treatment could be caused by differences in the rate/intensity of exercise performance (Ridgel et al., 2009). This hypothesis indirectly recalls the concept of voluntary versus forced exercise (FE), the latter being an exercise voluntarily achieved at a rate higher to that the subjects thought to be their maximum. To understand the differences in the effectiveness of voluntary exercise and FE, animal models of PD offer a theoretical framework. Indeed, only FE promotes motor behavioral recovery by modulating the expression of genes and proteins implicated in basal ganglia function (Zigmond et al., 2009; Tajiri et al., 2010). On the basis of these experimental results, Alberts et al. (2011) demonstrated that PD subjects who executed a lower-limb FE (stationary bicycle) exhibited a significant higher global improvement in motor symptoms than did patients who executed the same voluntary (not forced) training. The authors’ suggestion was that PD subjects may not be able to exercise (voluntarily) at adequately high rates to elicit brain adaptive changes. Neuroimaging studies confirmed that FE may actually trigger brain adaptive changes in PD (Beall et al., 2013; Alberts et al., 2016; Shah et al., 2016).

The present study is aimed to investigate the effect of an upper-limb exercise conceptually similar to the FE, mainly involving shoulder girdle and extensor head muscles, on motor and non-motor symptoms in PD. Exercise was based on our previously published methodology (Messa et al., 2016), in which participants exercised while seated on a specially designed device (Angel Wings^®^ device). A combined upper-limb motor training was used. The term FE pointed out that the learned exercise was voluntarily achieved at a rate higher to that which the PD patients thought to be their maximum. The Unified Parkinson Disease Rating Scale (UPDRS) scores, computerized dynamic posturography (by EquiTest equipment), fMRI, rs-fMRI, MRI brain perfusion by ASL, and neuropsychological tests were used before and after 2 months’ exercise intervention.

## Materials and Methods

### Subjects

Preliminarily, in order to assess postural instability and location of the COG, 28 PD patients in stage III according to the Hoehn and Yahr (1967) scale with “stooped” posture (forward head posture, rounded shoulders, scapular wide apart, and exaggerated lordosis of the lumbar spine) were studied with EquiTest (see below). The goal of dynamic posturography-based patient selection was to narrow the range of patients in order to obtain an almost homogeneous sample in terms of postural disability. On the basis of some results from EquiTest (composite sway and ESs), we enrolled 10 patients with idiopathic PD. Table 1 shows the demographic and clinical characteristics of the 10 subjects. Composite sway score was below 87% (the control mean being 81.4 ± 5.5%), and ES > 0.1 (the control range being −0.3 to 0.1). These fixed values were chosen so that all the selected patients had the ES and the location of the COG is similar and, at the same time, different from that of the healthy controls. The control group consisted of 16 healthy volunteers, age and sex matched (mean age 68 ± 7.2; seven females and nine males) with the enrolled PD subjects. The control group was selected only for computerized dynamic posturography analysis, with the purpose of establishing a cutoff limits for the initial selection of PD patients (see above).

**TABLE 1:** Demographic data and some clinical, EquiTest, and neurocognitive data of the 10 Parkinson subjects, before and after the training with Angel’s Wings.

		**Pretraining**	**Posttraining**	**3 weeks posttraining**	**6 weeks posttraining**	**8 weeks posttraining**
Age ± SD	69.1 ± 6.5					
Sex	3 F; 7 M					
Disease duration	5 years ± 1.3					
Duration of L-DOPA therapy	3 years ± 1					
L-DOPA dose		425 mg/day	425 mg/day	425 mg/day	425 mg/day	425 mg/day
Modified Hoehn and Yahr Scale		Stage 3	Stage 2.2^∗^	Stage 2.45	Stage 2.55	Stage 2.75
GDS score,		3.6 ± 2.2	2.2 ± 1.4^§^			
MDRS score		134 ± 6.3	137 ± 9.2^§^			
UPDRS III score		2.37 ± 0.43	0.85 ± 0.25^∗∗^	1.21 ± 0.32^∗∗^	1.6 ± 0.37^∗∗^	2.06 ± 0.51
Neck VAS score		5.1 ± 1.13	0.2 ± 0.4^∗∗^	1.85 ± 1.8^∗^	3.8 ± 1.9	4.4 ± 1.5
Shoulder VAS score		5.1 ± 1.2	0.55 ± 0.6^∗∗^	1.4 ± 0.9^∗∗^	3.55 ± 1.5^∗^	3.85 ± 1.4
Dorsal VAS score		2.55 ± 2.5	0.2 ± 0.6^∗∗^	0.3 ± 0.6^∗∗^	1.6 ± 1.6	1.9 ± 1.5
Lumbar VAS score		5.2 ± 2.6	0.9 ± 1.15^∗∗^	2.7 ± 2.3^∗^	3.9 ± 2.8	4.55 ± 2.5
Composite ES		74 ± 6.29	76.64 ± 6.2^§^			
COG Alignment		0.36 ± 0.16	0.21 ± 0.08^§^			
Composite MCT score (latency, ms)		148.2 ± 14.4	139 ± 15.77^§^			
WS backward (%)		94.71 ± 8.5	101.2 ± 7.7^§^			
WS forward (%)		95.78 ± 11.7	102.3 ± 10.6^§^			

*Data are reported as mean** ±** SD. COG, Center of Gravity; ES, equilibrium score; GDS, Geriatric Depression Inventory Scale; MDRS, Mattis Dementia Rating Scale; MCT, Motor Control Test; WS, weight symmetry. Mean UPDRS score is calculated as the mean of the single scores of the 14 items (ranging from 0: normal, to 4: unable to perform or complete). ^∗^*p* < 0.05; ^∗∗^*p* < 0.001. §Values reaching the statistical significativity with respect to pre-Angel’s Wings training condition. GDS score *p* = 0.029: MDRS score *p* = 0.018; composite ES: *p* = 0.02; COG: *p* = 0.01; MCT: WS forward: *t*(8) = 2.324, *p* = 0.03; WS backward: *t*(7) = 3.728, *p* = 0.007. Composite latency: *t*(8) = 3.375, *p* = 0.001.*

All selected patients had antiparkinsonian therapeutic regimen at a stable and optimized daily dose for no less than 4 weeks prior to taking part in present study. Primary exclusion criteria included cardiopulmonary or cerebrovascular disease, any type of dementia, visual impairment, and any medical or musculoskeletal contraindications to the exercise. Also, dyskinesia and painful off-state dystonia, Pisa syndrome, and scoliosis were excluded. MRI-incompatible metal implant, severe claustrophobia, and any pathological alteration demonstrated by MRI examination performed before treatment were considered exclusion criteria. Participants were instructed about the study; they signed a written informed consent prior to joining the study.

### Device and Exercise Training

**FIGURE 1:**
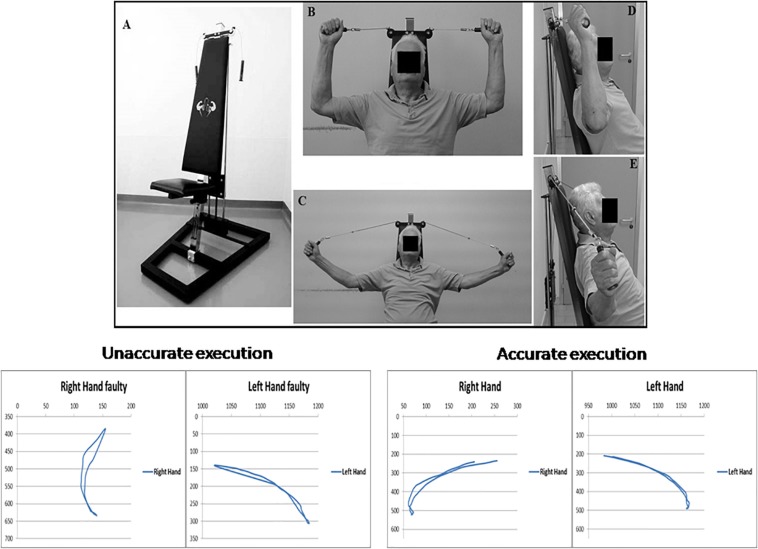
Top: Angel’s Wings device **(A)**; frontal **(B)** and lateral **(D)** views of the starting position of the exercise; frontal **(C)** and lateral **(E)** views of the distension of the forearms. At the bottom of the figure, on the right, are shown the trajectories (in pixels) of both hands required for a correct execution of the exercise with Angel’s Wings device, in an exemplificative patient. On the left are shown the trajectories obtained in the same subject before an appropriate period of learning of the exercise. A written, informed consent was obtained from the subject represented in the figure.

The Angel’s Wings (Figure 1A) is a patented device (patent number 0001401430) consisting of a seat (adjusted depending on the length of the user’s arms) and a system of cables and pulleys coupled to the adjustable weights (from 1 to 7 kg) that can be lifted by the subjects with two handles (Messa et al., 2016). Figures 1B–E outlines the task execution. Starting from a seated position (Figure 1B: frontal view; Figure 1D: lateral view), the subjects extends the forearms (Figure 1C: frontal view; Figure 1E: lateral view), keeping elbows at shoulder height, to lift a weight through the cables of the equipment. The forearms move in the frontal plane. The device recruited arm and axial–cervical–dorsal muscles, and thanks to the distribution of forces vectors, it mainly engages trapezius muscle (Messa et al., 2016). The exercise with the device needs some pretraining (usually two to three sessions) to be performed correctly. In fact, even though the exercise was apparently simple, it required a period of learning to reach an adequate quality of the performance (accuracy and rate). Although we did not measure learning, based on the observations of the trainers, it appeared that patients became skilled performers. It is significant that our patients moved relatively easily from cognitive (understanding) to the associative (or practice) stage, whereas they were unable to move to the autonomous (or automatic) stage of the skill learning process. As skill learning is a continuous and dynamic process that involves practice and time, we cannot assert that the latter stage is definitively precluded in PD patients.

At the bottom of the Figure 1, trajectory (in pixels) of both arms during movements is shown. Trajectories and duration were monitored by using the Microsoft Kinect (Clark et al., 2012). Each movement was 2 s in duration. The starting position is seated with the grab handles and palms facing the direction of the eyes. The spinal part of the rachis (including the neck) is outstretched, and it makes contact with the backrest, and the forearms are extended outward. In the positive phase of the movement, the weight, attached to the cables, is lifted perpendicularly, and during the return phase, it is brought back to its original location. By means of a system of pulleys, the weight always moves along a straight line perpendicular to the ground and on the same sagittal plane of the spine.

The training period was 8 weeks long for each patient, with two sessions of training per week. The exercise standard protocol consists in 16 sessions: twice a week (8 weeks in total), each session including five series of 2 min of exercise and 2 min of rest (60 repetitions for each series). It is important to underline that after learning the correct trajectories (reduction in errors and the development of smooth, effortless performance), spontaneous voluntary rate of patients was no more than 30–40 repetitions for each series. Therefore, in addition to the Microsoft Kinect monitoring, all series had to be carried out under the constant supervision of a trainer who guides and encourages the patients to perform the correct exercise at a constant, pre-established rate, ensuring a full and correct completion of the exercise for every subject. In addition, the trainer could easily understand, from the quality of the movements, if the single patient could push forward in his performance. The weights in the device were changed in every series starting with 2–3 kg in the first series, 4–5 kg in the second, 6–7 kg in the third, 4–5 kg in the fourth, and finally 2–3 kg in the fifth. The purpose of increasing and decreasing weights was to optimize muscle workout. Even the elderly patients could reach a full correct execution of the exercise with 6–7 kg of weight.

Our exercise and that of Alberts et al. (2011) overlap for the same basic principle: in both cases, the patients performed a similar aerobic resistance workout over what they thought was their maximum. Furthermore, the training strategy was also similar to that used by Alberts et al. (2011): in both cases, first, a warming-up phase, and an increase and finally a decrease in load were achieved. In other terms, a “pyramidal” training strategy was used in both studies.

Differently to the exercise proposed by Alberts et al. (2011), our exercise does not include an electrical/mechanical device that aid the patients on pushing beyond their limits; the latter purpose is indeed guaranteed by the trainer who guides the subjects during the exercise (see above). In this regard, from a conceptual point of view, our exercise is comparable with the FE.

Before and 7 days after the training, UPDRS-III score, visual analog scale (VAS) for pain, neuropsychological tests (MoCA and MDRS), Geriatric Depression Scale, SF-36, computerized dynamic posturography by EquiTest equipment, rs-fMRI, and ASL were assessed. UPDRS-III score and VAS were also used for the patients’ follow-up.

### Unified Parkinson Disease Rating Scale, Visual Analog Scale, and Neuropsychological Tests

Motor function was evaluated using the UPDRS Part III motor examination. VAS was stratified by body districts. UPDRS evaluation was performed according to methods previously validated by a PD expert neurologist (Vassar et al., 2012).

We administered a screening of neuropsychological test battery investigating general cognitive functions using the MoCA (Santangelo et al., 2015) and the MDRS (Mattis, 1988; Llebaria et al., 2008; Skorvanek et al., 2018), attention was extrapolated from the MDRS (Mattis, 1988; Skorvanek et al., 2018), quality of life was assessed by the SF-36 (Apolone and Mosconi, 1998), and depression was assessed by using the Geriatric Depression Scale (Yesavage et al., 1982).

UPDRS-III and VAS score results were verified at the end (7–10 days) and 3, 6, and 8 weeks after the end of the training. Data from UPDRS/VAS and neuropsychological test obtained before and after treatment were tested for normal distribution (D’Agostino and Pearson normality test [α = 0.05]). Parametric test was then used: repeated analysis of variance (ANOVA) measures with Tukey’s multiple-comparisons test for UPDRS/VAS values and *t*-test for paired data for neuropsychological test battery. The level of statistical significance was indicated by *p* < 0.05.

### Computerized Dynamic Posturography

Computerized dynamic posturography was evaluated by EquiTest (NeuroCom Int. Inc., Clackamas, OR, United States). The method was already described in detail (della Volpe et al., 2006). In summary, subjects stood barefooted with ankles directly above the *X*-axis of the force platform and feet equidistant from the *Y*-axis, their arms hanging loosely by their sides, facing the visual surrounding, and maintaining optic fixation straight ahead on a small cloud drawn at the center of the scenery. The subjects stood on the platform and attempted to maintain their balance in the Romberg position when sensorial conditions changed. A safety harness was used to prevent falls.

The SOT consisted of six conditions (SOT 1–6), each composed of three trials lasting 20 s. In conditions 1 and 2, subjects stood quietly with eyes open and closed, respectively. This ascertained if sway increases when visual cues are deleted and verified how effectively the participants made use of the somatosensory input. In condition 3, the subjects stood with the eyes open; the visual surround was sway referenced, and visual cues became inaccurate. In condition 4, the support surface was sway referenced; thus, somatosensory cues became inaccurate. Eyes closed and a sway-referenced support surface represent condition 5. This established how the participants made use of the vestibular cues when visual cues were removed and somatosensory cues were inaccurate. Finally, in condition 6, the visual surround and support surface were both sway referenced, which identified if the participants relied on visual cues even when they are inaccurate. The initial COG alignment is determined as the average antero-posterior COG location in the preceding half second before each SOT trial.

All six conditions were completed on the MCT, including three forward and three backward translations (small, 2.8°/s; and medium, 6.0°/s). During each translation, the horizontal displacement of the support surface is scaled according to the subject’s height. The trials were performed in a standardized order (first backward and then forward translations), and, if required, they were interrupted and a balance loss was recorded. The full MCT takes about 5–10 min to complete without rests. The MCT allows the software to compute the results for the following variables of interest: WS–weight bearing distribution in the left vs. right limbs prior to the onset of a translation as measured by the force transducers. The time lapse between the onset of support surface translation and the participant’s active force response was the latency; the latency was measured as the right and left feet averaged performance. Pre-exercise and post-exercise results (SOT and MCT) were statistically compared. D’Agostino and Pearson normality test (α = 0.05) was performed. Student *t*-test for paired data was used for MCT analysis. SOT data (ES and COG values) did not pass normality test, so the Wilcoxon matched-pairs signed rank test was used for these analyses. Statistical significance was *p* < 0.05.

### MRI Protocol and Analyses

MRI scans were performed on a Siemens Avanto scanner with a 12-channel head-coil. Pulsed ASL images were acquired using a PICORE Q2T sequence (TR = 3,100 ms, TE = 22 ms, TI1 = 700 ms, TI2 = 1,800 ms, number of slices = 16, thickness = 6.3 mm, gap = 1.6 mm, imaging matrix = 84 × 84, flip angle [FA] = 90°, and acquisition duration = 4 min 45 s). T1-weighted 3D-MPRAGE sequences (TR = 1,880 ms, TE = 3.38 ms, TI = 1,100 ms, FA = 15°, number of slices = 176, thickness = 1 mm, gap = 0 mm, and imaging matrix = 256 × 256) were used to obtain high-resolution T1-weighted axial images covering the whole brain. fMRIs were acquired using standard echo-planar imaging resting state (TR = 2,000 ms, TE = 20 ms, FA = 70°, number of slices = 37, thickness = 3.59 mm, gap = 4.64 mm, imaging matrix = 448 × 448, and acquisition duration = 8 min 36 s). To verify previous cerebrovascular events, only pretreatment MRI exam included sequences such as fluid-attenuated inversion recovery, diffusion-weighted imaging, and susceptibility weighted imaging.

#### Arterial Spin Labeling Data Analysis

Functional imaging data processing and analyses were performed using MATLAB R2014a (MathWorks, Natick, MA, United States)^1^, SPM12 (Wellcome Department of Cognitive Neurology, United Kingdom)^2^, and ASLtbx (Wang et al., 2008). *M*_0_ image consisted in the first volume of 91 ASL acquisitions; the residual 90 volumes were used as 45 control-label pairs (labeling first).

A six-parameter rigid body motion spatial transformation was used in aligning the raw echo-planar imaging time series. Then, the T1 images were co-registered to functional images. The spurious motion component caused by the systematic label/control alternation was regressed out from the motion parameters before applying the transformation on the images (Wang, 2012). In order to create 45 perfusion-weighted images, each tag and control pair were subtracted. These images were used to generate quantified maps of CBF by means of the software ASLtbx. Specifically, CBF was quantified as ml/100 g/min by Equation (1), as recommended by guidelines (Alsop et al., 2015).

(1) $$f=\frac{\Delta \,M\,\lambda \,R_{1\,\alpha }\,\exp\left(\omega \,R_{1\,\alpha }\right)}{2\,M_{0}\,\alpha }\,{\left[1-\exp\left(-\tau \,R_{1\,a}\right)\right]}^{-1}$$

In the equation, *f* is the CBF, *M* is the difference signal between the label acquisitions and control, *τ* represents the labeling time, α is the labeling efficiency, ω is the post-labeling delay time, λ is blood/tissue water partition coefficient, *R*_1α_ is the longitudinal relaxation blood rate, and *M*_0_ is approximated by the control image intensity (Warmuth et al., 2003).

Four-dimensional CBF images were masked to remove out-of-brain voxels and normalized to the Montreal Neurological Institute template in SPM12. The CBF images were processed using partial volume correction in the native ASL spaces and subsequently normalized to Montreal Neurological Institute space using a linear affine transformation. Then, to decrease noise for subsequent image subtraction, the images in the Montreal Neurological Institute space were spatially smoothed with a 6-mm full-width at half-maximum kernel. The smoothed, normalized CBF images were analyzed in SPM12 using a paired *t*-test with the within-subject factor “TIME” (two levels: before and after training). Age, gender, and days apart between pretreatment and posttreatment were entered as nuisance covariate. With the significance of the peak voxel (threshold *p* < 0.01) along with a stringent cluster size (100 voxels) taken into consideration, a permutation test for peak-cluster level error correction (AlphaSim) was used for whole-brain analysis (Song et al., 2011).

#### Resting-State Data Analysis

Functional image preprocessing and statistical analyses were carried out using SPM12 software and MATLAB 7.5 (MathWorks, Natick, MA, United States).

To allow for steady-state magnetization, the first three volumes of functional images were discarded for each subject. Echo-planar imaging images were slice-time corrected using the interleaved descending acquisition criteria and were realigned and re-sliced to correct for head motion using a mean functional volume derived from the overall fMRI scans. Subjects were excluded if the head motion exceeded 1.0 mm or rotation exceeded 1.0° for the period of the scanning. In order to obtain the better estimation of brain tissues maps, we implemented an optimized segmentation and normalization process using DARTEL (Diffeomorphic Anatomical Registration using Exponential Lie Algebra) (Ashburner, 2007) module for SPM12. In brief, this method is based on the creation of a customized anatomical template built directly from subjects’ T1-weighted images instead of the canonical one provided with SPM12. With this approach, it is possible to obtain a finer normalization into standard space; consequently, underestimation or overestimation of brain regions volume possibly induced by the adoption of an external template is avoided. In order to remove isolated voxels, the hidden Markov random field model was applied in all segmentation processes. Customized tissue prior images and T1-weighted template were smoothed using a 6-mm full-width at half-maximum isotropic Gaussian kernel. Functional images were then non-linearly normalized to standard space, and a voxel resampling to (isotropic) 3 × 3 × 3 mm was applied. Functional volumes were band pass filtered (0.01 < *f* < 0.08 Hz) to reduce low-frequency drift. To decrease the influence of the rising temperature of the MRI scanner, the linear trends were eliminated. Finally, a CompCor algorithm was used to manage physiological high-frequency respiratory and cardiac noise (Behzadi et al., 2007).

The smoothed, normalized resting-state images were analyzed using a paired *t*-test with the within subject factor “TIME” (two levels: before and after treatment) in MATLAB using the CONN toolbox^3^ (Whitfield-Gabrieli and Nieto-Castanon, 2012).

The Harvard-Oxford atlas (cortical and subcortical)^4^ (Desikan et al., 2006) was employed for resting-state functional connectivity analysis, which accounts for a whole-brain parcellation covering each cortical and subcortical region. Both a ROI analysis and a seed-to-voxel analysis were conducted in the second-level analysis to identify potential differences between before and after treatment. The ROI-to-ROI analysis allowed to pinpoint significant functional connectivity changes using two-sided comparison with a level of *p* < 0.05 false discovery rate corrected (Chumbley et al., 2010). Then, the most significant nodes were taken into account for seed-to-voxel analysis, which was performed using two-sided comparison with a voxelwise threshold level of *p* < 0.01 uncorrected and a cluster-level threshold of *p* < 0.05 false discovery rate corrected. Age, gender, and day apart between pretreatment and posttreatment were entered as second-level nuisance covariate.

## Results

### Unified Parkinson Disease Rating Scale, Visual Analog Scale, and Neuropsychological Tests

Table 1 shows the UPDRS-III mean score. There was a statistically significant improvement at the end of the training (points 68% improvement with respect to pretraining). Percent improvement was 48, 32, and 13% at 3, 6, and 8 weeks after the end of the treatment, respectively. ANOVA test: *F*(1,69) = 133.5, *p* < 0.0001; significative data from Tukey’s multiple-comparisons test: pre vs. post, pre vs. 3 weeks, and pre vs. 6 weeks (for all: *p* < 0.0001). No significant changes were found 8 weeks after the end of the treatment. VAS mean score is shown in Table 1. Neck, shoulder, dorsal, and lumbar pain was significantly reduced posttraining, recovering pretraining intensity 5 weeks (neck and lumbar spine) and 8 weeks (shoulder and dorsal spine) after the end of exercise. VAS score at the neck: ANOVA test: *F*(1,54) = 30.25, *p* < 0.0001; significative data from Tukey’s multiple-comparisons test: pre vs. post (*p* < 0.0001) and pre vs. 3 weeks (*p* = 0.0017). VAS score at the shoulder: ANOVA test: *F*(1,54) = 46.84, *p* < 0.0001; significative data from multiple-comparisons test: pre vs. post (*p* < 0.0001), pre vs. 3 weeks (*p* < 0.0001), and pre vs. 6 weeks (*p* = 0.013). VAS score at the dorsal area: ANOVA test: *F*(1,54) = 7.1, *p* = 0.0002; significative data from Tukey’s multiple-comparisons test: pre vs. post (*p* = 0.035) and pre vs. 3 weeks (*p* = 0.03). VAS score at the lumbar area: ANOVA test: *F*(1,54) = 10.4, *p* < 0.00001; significative data from Tukey’s multiple-comparisons test: pre vs. post (*p* = 0.0002) and pre vs. 3 weeks (*p* = 0.024).

With regard to neuropsychological assessment, the baseline evaluation showed lower-than-normal scores on the test of general cognitive functions at MDRS (mean = 134 ± 6.3; the cutoff is 137), the most sensitive scale to cognitive impairment in PD, because it investigates the functions most compromised in this disease, such as frontal functions (Skorvanek et al., 2018). Pre-exercise training, the average scores of the neuropsychological tests were the following: 24.42 ± 3.26 for the evaluation of general cognitive functions carried out through MoCa; 3.6 ± 2.2 for the depression (scale Geriatric Depression Inventory Scale); 155.44 ± 633.29 for the variable of mental functioning (Mental Component Summary) obtained from the sum of the subscales of SF-36; 175.06 ± 21.24 for the variable of physical functioning (Physical Component Summary); and 35.9 ± 0.9 for the subscale of attention obtained from MDRS.

At the analysis carried out on the variables considered, three main effects emerge between pre-exercise and post-exercise training periods. There is a significant difference [*t*(9) = 2.8053; *p* = 0.0205] in the average Geriatric Depression Inventory Scale score, from 3.6 ± 0.22 to 2.2 ± 1.4, indicative of a reduction of depressive status posttraining; the confidence interval (0.27–2.53) contains 95% of confidence of the real difference between the Geriatric Depression Inventory Scale score before and after treatment. There is also a significant difference [*t*(8) = −2.4947; *p* = 0.029] between the mean MDRS score (from 134 ± 6.3 to 137 ± 9.2), most likely an index of improvement of general cognitive functions; the confidence interval (0.45–7.21) contains 95% of confidence of the real difference between the MDRS score pretraining and posttraining. Finally, a significant difference [*t*(8) = −2.863; *p* = 0.018] emerges between the average attention (from 35.9 ± 0.9 to 36.8 ± 0.4), indicative of an improvement in the performance of patients in the attention tests at the second measurement; the confidence interval (0.19–1.61) contains 95% of confidence of the real difference between the pretraining and posttraining attention scores. The results showed no significant difference between the average score at the first and second evaluations in the other tests (MoCA; Mental Component Summary; Physical Component Summary).

### Computerized Dynamic Posturography

**FIGURE 2:**
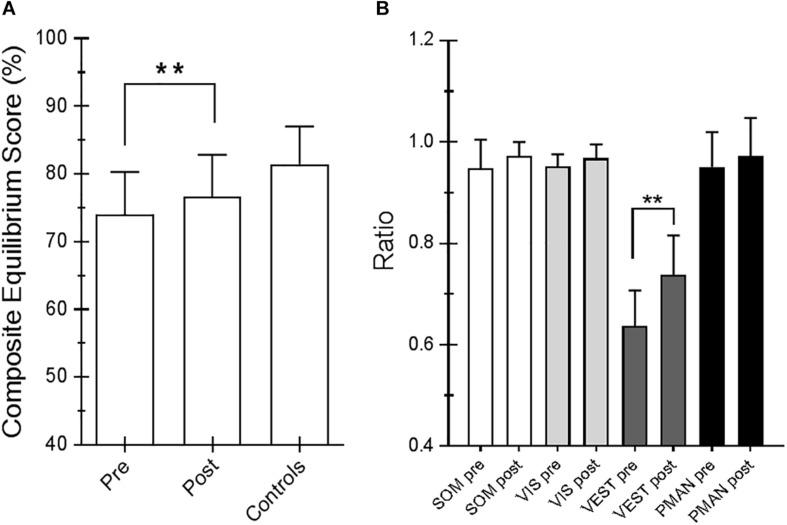
**(A)** Equilibrium score (ES) composite pretraining and posttraining with Angel’s Wings and the control values. **(B)** Somatosensory ratio composite pretraining and posttraining with Angel’s Wings and in the controls subjects. “SOM” refers to the sway increase when visual information is removed; low scores: poor use of somatosensory references. “VIS” refers to the sway increase when somatosensory information is removed; low scores: poor use of visual references. “VEST” refers to the sway increase when visual information is removed, and the somatosensory information is incorrect; low scores: poor use of vestibular information or no vestibular information. Statistical analysis is performed between pretraining and posttraining only in Parkinson’s disease (PD) subjects. Values are reported as mean and standard deviation. ^∗∗^*p* = 0.02 (Wilcoxon matched-pairs signed rank test).

**FIGURE 3:**
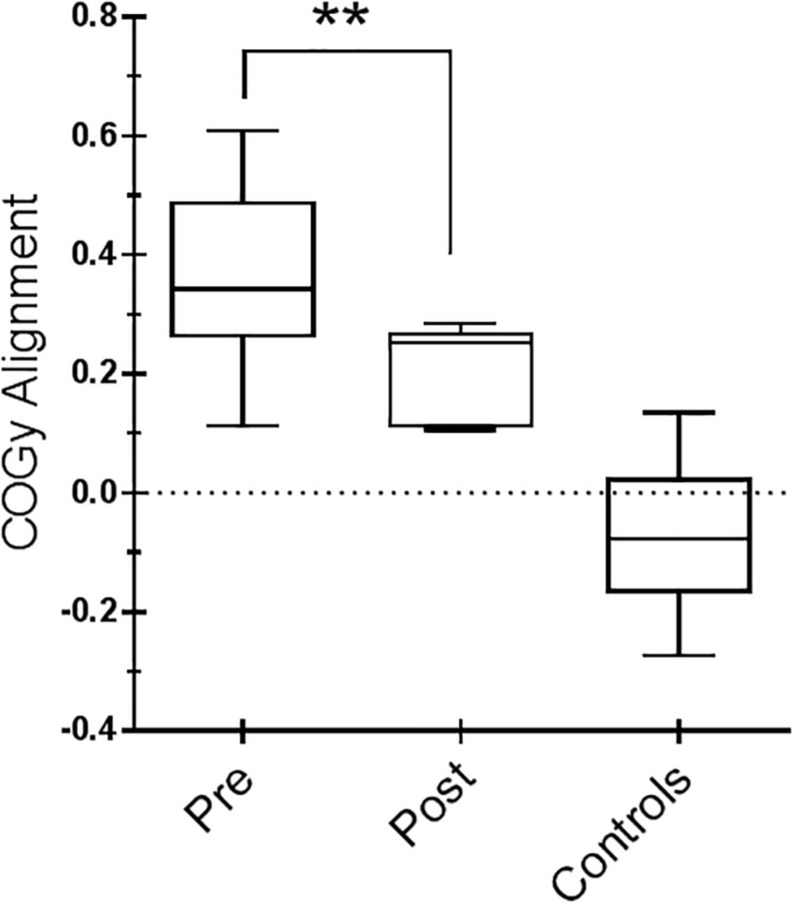
Center of gravity (COG) alignment prior to the onset of the Sensory Organization Test (SOT) conditions and control values. Box and whiskers represent minimum, maximum, median, and 25th and 75th percentiles. Posttraining, a more accurate alignment of the center of gravity relative to the base of support is shown. Statistical analysis is performed between pretraining and posttraining only in Parkinson’s disease (PD) subjects. ^∗∗^*p* = 0.01 (Wilcoxon matched-pairs signed rank test).

**FIGURE 4:**
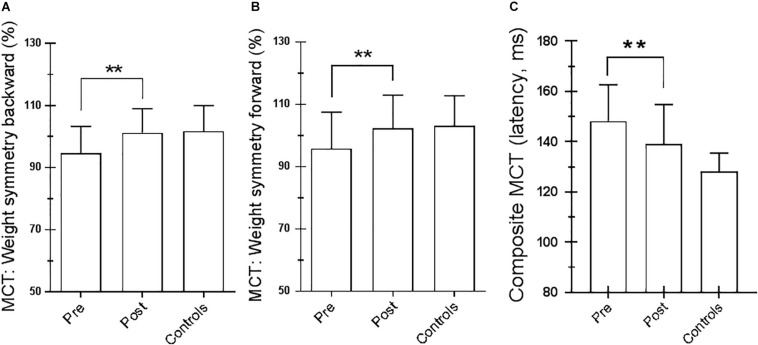
Motor control test (MCT). **(A)** Weight symmetry (WS) in backward pretraining and posttraining; ^∗∗^*p* = 0.007 (paired *t*-test). **(B)** WS in forward pretraining and posttraining; ^∗∗^*p* = 0.03 (paired *t*-test). **(C)** Averaged latencies of the right and left feet. ^∗∗^*p* = 0.001 (paired *t*-test). Statistical analysis is performed between pretraining and posttraining. Values are reported as mean and standard deviation. The third column of each figure represents the control values. Statistical analysis is performed between pretraining and posttraining only in Parkinson’s disease (PD) subjects.

For the SOT, we computed the results for the ES, an overall indicator of balance, and the COG alignment prior to onset of each trial. With regard to the ES, the posttraining values of conditions SOT 1, 5, and 6 were significantly different from those obtained pretraining (*p* = 0.019 [*W* = 45], *p* = 0.013 [*W* = −54] and *p* = 0.018 [*W* = −52], respectively) in PD subjects. Figure 2 shows ES composite (A) and the somatosensory ratio (B). Composite subscore of the PD subjects was significantly lower in pretraining with respect to posttraining (see values in Table 1). The sensory analysis ratios used in combination with the ES to identify specific impairments of the individual’s sensory system show a significant improvement in vestibular values (see Figure 2B). Figure 3 illustrates mean COG alignment during the six conditions: at posttraining, there is a significant backward shift of COG relative to pretraining analysis (see values in Table 1). Figure 4 shows MCT analysis. WS values during graded backward and forward translations reveal that prior to the onset of the support surface translation, in the posttraining period, the participant had their weight relatively centered between the left and right legs (Figures 4A,B). In Table 1, the values of the WS analysis are reported. The sketch in Figure 4C relative to the mean latency shows that the time lapse between onset of the perturbation and postural correction is significantly reduced with respect to the pretraining (see Table 1 for the values).

### Magnetic Resonance Imaging

#### Changes in Cerebral Blood Flow

**FIGURE 5:**
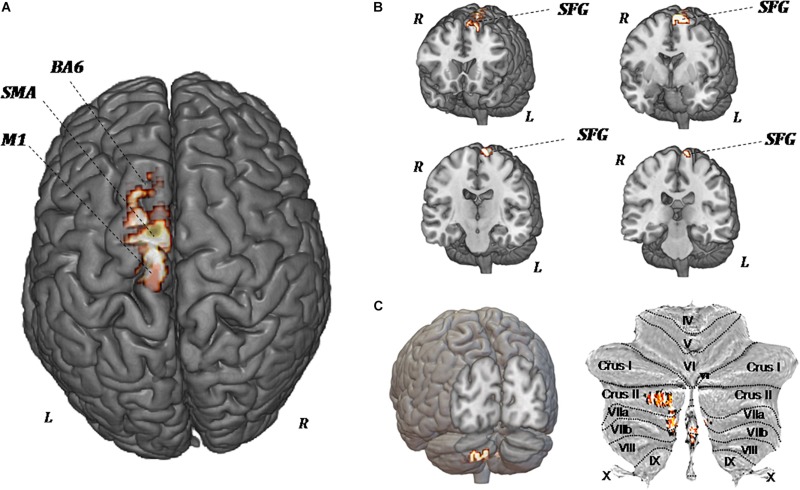
MR perfusion maps demonstrate pre-forced exercise vs. post-forced exercise cerebral blood (CBF) flow changes. The red areas, overlaid on anatomical volumetric T1-weighted images axial **(A)** and coronal views **(B,C)**, represent the voxels with a statistical significant CBF augmentation: the left superior frontal gyrus and left cerebellar hemisphere. Comparing the CBF values of each voxels between pretreatment and posttreatment, in all patients, the increased CBF areas are observed in the left supplementary motor area (SMA), left premotor area (BA6), primary motor area (M1), and left cerebellum (crus II, VIIa, and VIIb).

Figure 5 shows the significant regional differences in CBF observed after upper-limb training, specifically in the left superior frontal gyrus (*k* = 296; −8, −1, 74), left SMA (*k* = 191; −2, −12, 58), left premotor area (*k* = 160; −10, −20, 78), M1 (*k* = 103; −13, −22, 76), and left cerebellum (*k* = 140; −2, −70, −44).

Because of the heterogeneity of function and anatomy of the cerebellum, a previously validated parcellation atlas was utilized to compare the voxel-level values before and after training. Significant clusters were mapped on the anatomical cerebellum parcellation by Diedrichsen et al. (2011). Overall, significant changes seem to be exclusively related to the medial part of cerebellum, specifically crus II, VIIa, and VIIb (Figure 5C).

#### Changes in Resting-State Functional Connectivity

**FIGURE 6:**
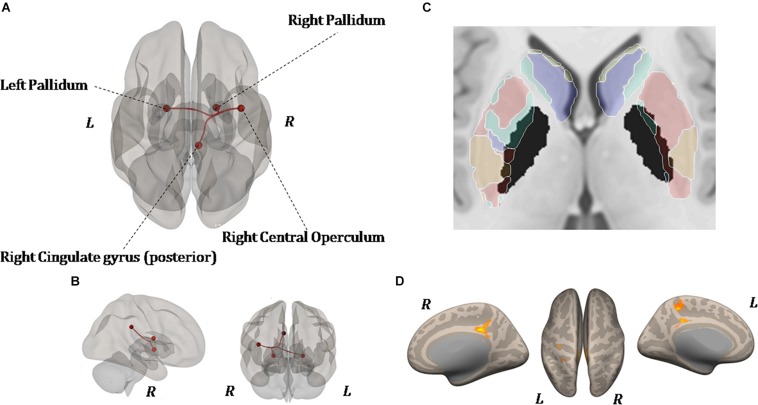
Resting-state functional connectivity differences. Functional image processing and statistical analyses demonstrated increased cortico-subcortical connectivity with respect to pretraining stage. In **(A,B)** the region of interest (ROI)-to-ROI analysis shows significant functional connectivity changes: both globus pallidi show increased connection to the right central operculum, right cingulate gyrus posterior, and left sensorimotor cortex. In **(C,D)**, seed-to-voxel analysis (left and right globus pallidi as seed) demonstrates a functional connectivity between the left primary motor cortex and left superior frontal gyrus. In **(D)**, the colored areas overlaid on flattering images of the brain represent the areas of cortical connectivity.

As shown in Figure 6, ROI-to-ROI analysis for rs-fMRI after training reveal significant increased positive correlations in the right central operculum (*t* = 4.65, *p*-uncorrected = 0.003, *p*-false discovery rate = 0.02), posterior right cingulate gyrus (*t* = 4.43, *p*-uncorrected = 0.005, *p*-false discovery rate = 0.04), and left (*t* = 4.24, *p*-uncorrected = 0.0004, *p*-false discovery rate = 0.03) and right (*t* = 4.90, *p*-uncorrected = 0.0001, *p*-false discovery rate = 0.01) pallidum. Given that the relationship between PD and the globus pallidus is well documented, the left and right pallidi were taken into account for seed-to-voxel analysis, where pretraining and posttraining comparison showed a stronger connectivity with four big clusters of voxels in the posterior cingulate cortex (*k* = 1041; 6, −42, 14), the left postcentral and precentral gyri (*k* = 513; −18, −44, 50), the left frontal orbital cortex (*k* = 324; −24, 12, −16), and the precuneus (*k* = 274; 8, −62, 40). All regions with significant connectivity changes are listed in Table 2.

**TABLE 2:** Anatomical region of interest with significant connectivity changes.

**Anatomical region (ROI)**	**Cluster**			**Cluster distribution**	***K***	***p*-FDR**	***p*-uncorrected**	**Increase/decrease connectivity**
	***x***	***y***	***z***					
Pallidum (Land R)	6	−42	14	525 voxels cingulate gyrus, posterior division	1,041	0.000073	0.0000001	↑
				159 voxels precuneus cortex				
				46 voxels intracalcarine cortex right				
				28 voxels brain stem				
				27 voxels supracalcarine cortex R				
				18 voxels vermis 3				
				10 voxels lingual gyrus L				
				6 voxels vermis 4–5				
Pallidum (L and R)	−18	−44	50	134 voxels postcentral gyrus L	513	0.003419	0.000026	↑
				27 voxels precuneus cortex				
				16 voxels precentral gyrus L				
Pallidum (L and R)	−24	12	−16	54 voxels insular cortex L	324	0.028998	0.000333	
				41 voxels frontal orbital cortex L				↑
				18 voxels subcallosal cortex				
				17 voxels putamen L				
				6 voxels frontal pole L				
				3 voxels cingulate gyrus, anterior division				
				2 voxels frontal operculum cortex L				
				2 voxels caudate L				
				2 voxels accumbens L				
Pallidum (L and R)	8	−62	40	208 voxels precuneus cortex	274	0.047469	0.000727	↑
				28 voxels cuneal cortex R				
				5 voxels lateral occipital cortex, superior division R				

*FDR, false discovery rate; L, left; R, right.*

**FIGURE 7:**
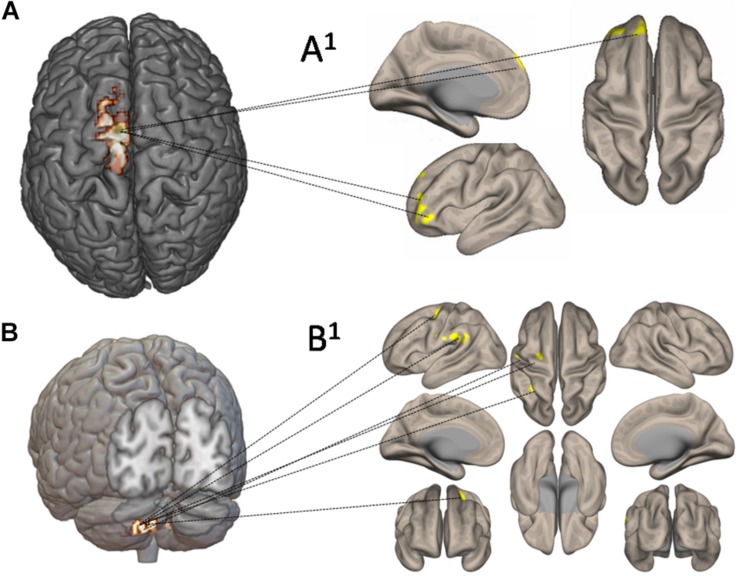
Functional connectivity and cerebral blood flow (CBF) changes by using seed-to-voxel analysis. In **(A)**, increased CBF areas in the left supplementary motor area and primary motor cortex have been used as seed: the functional connectivity is demonstrated with frontal lobe (area 10) **(A1)**. In **(B)**, increased CBF areas in left crus II have been used as seed: the functional connectivity has been demonstrated with the left prerolandic and post-rolandic areas and the left supramarginal area **(B1)**.

Figure 7 shows the functional connectivity and CBF changes by using seed-to-voxel analysis. When increased CBF areas in the left SMA and M1 have been used as seed, the functional connectivity is demonstrated with frontal lobe (area 10) (Figures 7A,A1). When increased CBF areas in the crus II (left cerebellum) have been used as seed, the functional connectivity is demonstrated with the left pre-rolandic/post-rolandic areas and left supramarginal area (Figures 7B,B1).

## Discussion

Experiments on FE in PD models demonstrated neuroprotective effects or enhancement of the neuronal differentiation with subsequent global improvement in deteriorated motor function (Tillerson et al., 2003; Tajiri et al., 2010). These models offer a hypothetical framework for understanding differences in the efficacy of forced versus voluntary exercise. In fact, dramatic effects of conventional voluntary exercises have not been reported in PD exercise trials, although they may transiently improve physical functions and health-related quality of life (meta-analysis by Goodwin et al. (2008)). On the contrary, PD patients undergoing an assisted bicycle pedaling exercise with the cycling rate controlled to be over the voluntary pedaling level (i.e., an FE) lead to global clinical improvements on PD symptoms (Alberts et al., 2011; Stuckenschneider et al., 2015). Interestingly, when comparing under the same aerobic intensity exercise voluntarily achieved at a rate corresponding to that the PD patients thought to be their maximum with that achieved at higher rate, only PD patients following FE exhibited a significant global clinical improvement (Alberts et al., 2011). Some further pilot studies failed to confirm a global clinical improvement in PD patients after FE; for example, Qutubuddin et al. (2013) did not show any relevant clinical effect by using a stationary bicycle with motorized pedals. However, as the authors stated, the capacity of the PD subject to maintain sufficient exercise intensity to produce appreciable benefit to the patient represents one important limiting factor. Levy-Tzedek et al. (2018) studied PD patients after execution of large, fast-paced rhythmic movements of the upper limb without instructions by the experimenter. They found improvement in the upper-limb UPDRS motor score but no a global clinical effect.

In the present study we used the upper-limb training in a sitting position. This training conceptually corresponds to an FE, since the learned exercise had to be achieved at a rate higher to that of the PD patients spontaneously thought to be their maximum, thanks to the continuous guidance of a trainer (see Materials and Method session).

### Unified Parkinson Disease Rating Scale, Visual Analog Scale, and Neuropsychological Score

After 2 months’ upper-limb training, we documented a significant improvement in the UPDRS-III and VAS score, along with an improvement of the neurocognitive tests with respect to the pretraining. UPDRS-III score improved by 68% when compared with baseline and returned to pretraining values 8 weeks after the end of training, documenting a global, long-lasting improvement in PD motor symptoms. Pain score exhibited a very similar time course with a minimum after the end of training, recovering to pretraining values 6 weeks after the end of treatment. These clinical changes were matched by a general improvement of cognitive functions of the patients, testified by the amelioration of the performance in the attention tests and by a reduction of depressive symptomatology. Our data are in line with the notion that rehabilitation strategies targeting one factor could lead to the improvement of the other, in particular enhancements in cognition (Crowley et al., 2018; Intzandt et al., 2018).

### Exercise and Postural Balance

All PD patients enrolled in the present study had moderate/severe balance and postural abnormalities (flexed trunk posture), both while moving (dynamic balance) and while standing still (static balance). After 8-week upper-limb training, they exhibit significant improvement in balance and postural control as revealed by SOT and MCT: (1) better alignment of the gravity center; (2) improved backward and forward WS; and (3) improvement of anticipatory motor strategies. This suggests that intrinsic postural motor learning is preserved in PD patients. In PD subjects, the ability to partially recovery balance and posture control can result from re-weighting the contributions of areas implicated in the central postural motor learning network (e.g., the medial frontal cortex, medullary locomotor areas, or cerebellum), to compensate for dysfunction in cortico-basal networks (Jahn et al., 2008; Fling et al., 2013).

To control posture-regulating muscles in the whole body, posture control strategies require visual and vestibular inputs, as well as both tactile and proprioceptive somatosensory inputs, principally in trunk and legs (Horak and Macpherson, 1996). The most relevant post-exercise change in sensory input of our PD patients was an enhanced ability to use vestibular information. In fact, in a condition of postural stability when standing on an unstable surface with closed eyes, vestibular information dominates over the other sensory inputs (Cenciarini and Peterka, 2006). It is plausible that vestibular information was basically altered/underused in patients with PD, although their vestibular apparatus is usually normal (Frenklach et al., 2009). As animal and human studies have demonstrated that vestibular signals give a relevant contribution to process egomotion information and to control posture and equilibrium (Cronin et al., 2017), it is possible that vestibular dysfunction may contribute to the postural instability of PD subjects (Allum et al., 2011). The recovery of vestibular function observed in our PD patients cannot be attributed to some vestibular “rehabilitation” because, under our training condition (upper-limb exercise in a sitting position), no vestibular stimulation occurred. Our explanation is an increased gain of vestibular signals due to enhanced or rebalanced proprioceptive input from neck muscles as a consequence of the upper-limb exercise (Messa et al., 2016). Evidence exists that the vestibular-evoked perception of body rotation is enhanced by neck-proprioceptive input (Panichi et al., 2011). In fact, the cerebellar interpositus nucleus integrates vestibular and neck proprioceptive signals. In addition, it is known that this cerebellar nucleus specifically targets limb movement premotor regions that utilize sensory signals to regulate movement (Houk et al., 1993; Miller et al., 1993). Recently, Luan et al. (2013) identified a population of cerebellar interpositus neurons with a precise matching of vestibular and proprioceptive signals, which activated the otolith organs and semicircular canals and involved both flexion and rotation in the spine. Such neurons code body motion in space, which may be the proper platform for governing the movements of the limb.

### Exercise and Neuroimaging

Extensive neuroimaging studies during different tasks-evoked fMRI in PD patients mainly found hypoactivation of the SMA and hyperactivation of the cerebellum, premotor area, and parietal cortex with respect to healthy subjects (Pan et al., 2017). However, as pointed out by Turner et al. (2003), abnormalities of brain activity in PD patients, as deduced from BOLD signal, may vary by varying the motor task. For this reason, the analysis under resting-state conditions could offer more reliable results. Briefly, by using resting-state BOLD correlations on a voxel-by-voxel basis in fMRI, in addition to disconnection of corticostriatal circuits, PD patients exhibited decreased functional connectivity between the hemispheres (Luo et al., 2015) and between the globus pallidus and cerebellum (Hacker et al., 2012), sensorimotor cortex (Baudrexel et al., 2011), and SMA (Kurani et al., 2015).

On the contrary, increased functional connectivity has been observed between the subthalamic nucleus and motor cortex (four and six areas) (Baudrexel et al., 2011). However, a relevant point is that changes in functional connectivity may be contingent upon the duration, severity, and treatment of the disease (Engels et al., 2018).

#### Cerebral Blood Flow (Arterial Spin Labeling)

In the present study, we have analyzed brain changes under resting-state condition before and after 8 weeks of upper-limb exercise training. Seven to 10 days after the end of training, CBF analysis by ASL showed a regional increase of CBF in the left M1, left SMA, and left cerebellar cortex (crus II), with all structures paying a pivotal role in posture and motor control (Takakusaki, 2017).

From classical perspective, the M1 cortex acts as the final cortical output for already processed movement commands, relaying signals from premotor cerebral cortical sites to the spinal cord. However, evidence exists of more multifaceted and central roles for the M1 in processing motor-related information (Omrani et al., 2017). Functions classically attributed to the SMA include its contribution to postural stabilization, to internally generated movements, to coordination of the hands, and to planning of the sequences of the movement. Convergent data from different scientific approaches strongly suggest that the most phylogenetically recent section of the cerebellum, principally the crus I and II, make contributions to parallel cortico-cerebellar loops implicated in a variety of aspects including cognition (Schmahmann, 2004; Habas et al., 2009).

A study about the evolution of the cerebellar cortex showed that the crus I and crus II cerebellar sections were significantly larger in humans compared with chimpanzees and capuchins. Indeed, Balsters et al. (2010) suggested that the cortical motor areas might entrain representations of motor memory in connected cerebellar parts and utilize these to execute learned movements. The prefrontal cortex might similarly entrain plastic circuitry in crus I and crus II to store representations that might be deployed during skilled cognitive operations (Hayter et al., 2007; Balsters and Ramnani, 2008). Furthermore, Yamaguchi and Sakurai (2016) observed in rats that inactivation of the cerebellar cortex, crus II, disrupted accurate temporal prediction of learned temporal intervals, demonstrating that it plays a role in rapid temporal processing. As suggested by Wu and Hallett (2005), the increased CBF of left crus II, observed in our patients, could reflect a mechanism of functional compensation for defective basal ganglia.

Increased CBF in the M1, SMA, and cerebellum has been also shown in healthy subjects during steady-state cycling exercise, but it reduced to initial values at the end of exercise (Hiura et al., 2014). On the contrary, in our patients, increased CBF was still present 7–10 days after the end of FE training.

#### Resting-State fMRI

Seven to 10 days after the end of the upper-limb exercise, functional image processing and statistical analyses demonstrated increased cortico-subcortical connectivity with respect to pretraining stage. Specifically, the bilateral globus pallidus showed increased connection to the right central operculum, right cingulate gyrus posterior, and left sensorimotor cortex (see Table 2). Also, seed-to-voxel analysis demonstrated a functional connectivity between the left M1 and left superior frontal gyrus (area 10). Left crus II showed strengthened connections with the left pre-rolandic area, left post-rolandic area, and left supramarginal area. The functional implication of these strengthened connections after the exercise is not obvious, but the possibility exists that it may represent an FE-induced brain adaptive and/or compensatory mechanism.

Alberts et al. (2016), by using task-evoked fMRI (bilateral finger-tapping task), showed a higher patterns of activation in the M1 and SMA, thalamus, and basal ganglia in PD after a lower-extremity FE training (i.e., assisted bicycle pedaling). These data are to a certain extent in line with our findings in resting condition, although a direct comparison cannot be made because they used a motor task to monitor brain imaging and the cerebellum was excluded from their analysis. It is, however, of interest that the general direction of brain functional connectivity changes after FE training seems to be similar to that produced by medicaments (Alberts et al., 2016).

## Conclusion

Animal models of PD offer a rationale and theoretical framework for the utilization of an FE intervention in PD. Indeed, decreased motor activation may limit PD patients’ ability to generate voluntary movements at rates necessary to improve global motor functioning. The development of a non-medicament and non-surgical therapeutic approach to ameliorate the motor function would provide an attractive adjunct to available PD treatment methods. We have developed an upper-limb exercise paradigm to augment the voluntary movements of PD patients to assist them in safely achieving an exercise rate greater than their voluntary rate. Our results indicate that PD patients completing an 8-week upper-limb exercise intervention exhibited a significant improvement in clinical motor rating scores as well as in static and dynamic postural stability. Furthermore, CBF analysis and functional connectivity have shown widespread post-exercise changes in the activation pattern with respect to baseline, involving the SMA, M1, basal ganglia, and cerebellum.

As mentioned in the Section “Discussion,” all the brain structures subjected to the observed post-exercise change are closely involved in the control of standing and locomotion. Although this does not allow us to conclude that actually they are the neuronal bases of clinical improvement in our patients, a sure conclusion can be proposed: the brain of subjects with PD can still be switched toward function adaptation, independently from drug therapy. This strengthens the rationale for including structured exercise programs as part of a patient’s therapeutic regimen.

The present study extend the results of Alberts et al. (2011, 2016) adding the evidence that the upper-limb exercise executed beyond the patient’s limits may give clinical global advantage in PD patients. This result may not be surprising because a clear-cut bidirectional coupling between upper and lower limbs has been also documented in humans. In particular, commands from the control centers regulating arm movement are given access to the lower limbs. In such way, the contribution from the upper limbs is functionally gated to support the action of the legs (Balter and Zehr, 2007; Huang and Ferris, 2009). Indeed, elements of quadrupedal interlimb coordination are conserved in humans (Dietz, 2002). A limitation of our study is the relatively small sample size. On the other hand, we have adopted extreme selection criteria (see “Subjects” section) in order to obtain a population as homogeneous as possible from a clinical and instrumental point of view. Finally, the recording of a variety of behavioral data represents a strength of the study because it can help to analyze the therapeutic effect of the exercise paradigm from different dimensions.

## Data Availability Statement

The datasets generated for this study are available on request to the corresponding author.

## Ethics Statement

Informed consent was obtained from all subjects prior to testing, and ethical approval for the study was obtained from the AOUS of Siena. The protocol was executed in accordance with the Declaration of Helsinki.

## Author Contributions

LM, FG, and AR conceived the research. DC, DM, BP, and CB preprocessed the data. CB, DC, LM, and DM analyzed the data. AR, LM, and FG prepared the figures and drafted the manuscript. AR, LM, FG, and ES edited and revised the manuscript.

## Conflict of Interest

The authors declare that the research was conducted in the absence of any commercial or financial relationships that could be construed as a potential conflict of interest.

## Footnotes

1 www.mathworks.com

2 www.fil.ion.ucl.ac.uk/spm

3 http://www.nitrc.org/projects/conn

4 www.fmrib.ox.ac.uk/fsl/

## Abbreviations

ASL: arterial spin labeling
CBF: cerebral blood flow
COG: center of gravity
ES: equilibrium score
fMRI: functional magnetic resonance imaging
M1: primary motor area
MCT: motor control test
MoCA: Montreal Cognitive Assessment
MDRS: Mattis Dementia Rating Scale
PD: Parkinson’s disease
ROI: region of interest
rs-fMRI: resting-state fMRI
SF-36: Health Survey Short Form-36 scales
SMA: supplementary motor area
SOT: Sensory Organization Test
UPDRS: Unified Parkinson’s Disease Rating Scale
WS: weight symmetry.
